# Long noncoding RNA NNT-AS1 promotes hepatocellular carcinoma progression and metastasis through miR-363/CDK6 axis

**DOI:** 10.18632/oncotarget.21321

**Published:** 2017-09-28

**Authors:** Ye-Bin Lu, Qin Jiang, Man-Yi Yang, Ji-Xiang Zhou, Qi Zhang

**Affiliations:** ^1^ Department of General Surgery, Xiangya Hospital, Central South University, Changsha, 410013, China; ^2^ Department of Ultrasonography, Xiangya Hospital, Central South University, Changsha, 410013, China; ^3^ National Hepatobiliary and Enteric Surgery Research Center, Xiangya Hospital, Central South University, Changsha, 410013, China; ^4^ Department of Hepatobiliary and Pancreatic Surgery, Xiangya Hospital, Central South University, Changsha, 410013, China

**Keywords:** hepatocellular carcinoma, long non-coding RNA, NNT-AS1, miR-363, CDK6

## Abstract

Long non-coding RNAs (lncRNAs) have been tested to act as important regulator in liver cancer genesis and progression. LncRNA Nicotinamide Nucleotide Transhydrogenase-antisense RNA1 (NNT-AS1) has been reported to participate in the tumorigenesis. However, the exact molecular mechanism of NNT-AS1 in hepatocellular carcinoma (HCC) is still unknown. In present study, our team identified the up-regulated expression of NNT-AS1 in HCC tissue and cell lines compared with adjacent noncancerous tissue and normal cells. Moreover, HCC patients with high NNT-AS1 levels had poor prognosis than that with low NNT-AS1 level (p=0.0089). *In vitro*, gain- and loss-of-function experiments revealed that enhanced NNT-AS1 expression promoted the proliferation ability and alleviated the cycle arrest and apoptosis, while NNT-AS1 knockdown suppressed the proliferation and induced G0/G1 phase arrest and apoptosis. *In vivo*, NNT-AS1 knockdown inhibited the HCC neoplastic tumor volume and weight. Bioinformatics analysis and luciferase reporter assay validated that miR-363 targeted NNT-AS1 and CDK6 3’-UTR. MiR-363 was down-regulated in HCC tissue and cells. NNT-AS1 competed with CDK6 for miR-363 binding and could increase CDK6 expression. In summary, our results suggest the oncogenic role of NNT-AS1 in HCC tumorigenesis through miR-363/CDK6 axis, providing a novel therapeutic target for human HCC.

## INTRODUCTION

Hepatocellular carcinoma (HCC) is one type of the most common malignant tumors, accounting for the second leading cause of worldwide cancer-related deaths with an increasing incidence annually [1, 2]. Although the examination methods and therapeutic methods for HCC is upgrading, the overall survival rates of HCC patients are still very poor [3]. Moreover, the underlying molecular and biological functions correlated with HCC are still unclear. Consequently, it has great challenge to investigate novel biomarkers and therapeutic targets for early detection of HCC [4].

Long noncoding RNAs (lncRNAs) are a class of non-protein coding RNA transcripts longer than 200 nucleotides [5]. Emerging evidences have illustrated that endogenous lncRNAs in eukaryocyte participate in the transcription and post-transcription regulation, acting crucial regulator in the tumorigenesis [6]. With the development of high-throughput RNA sequencing and bioinformatics analysis, numerous newfound lncRNAs have been reported to act as oncogenes or tumor suppressors in multiple oncogenesis progression. For instance, lncRNA SNHG1 is remarkably upregulated in HCC tissues and cell lines, and promotes the HCC cell proliferation, invasion and migration through inhibiting miR-195 [7]. LincRNA-p21 is downregulated in HCC tissue and cells, and the enhanced expression of lincRNA-p21 could suppress Notch singling and epithelial-mesenchymal transition [8].

For decades, lots of researches have extensively elucidated the expression profiles and functions of microRNAs (miRNA) in series of diseases, involving all the cancers. MiRNAs are a type of non-coding RNA (ncRNA) with 18-22 nucleotides [9]. The regulatory mode of miRNAs is to directly bind with the 3’-UTR of target genes mRNA and negatively regulate the mRNA and functional protein expression. Thus, the regulation of miRNAs belongs to post-transcriptional level modulation [10]. The pathogenesis of HCC is complex and hundreds of miRNAs have been reported to participate in the occurrence and development progression [11]. MiR-363 has been tested as tumor suppressor in several cancers, such as gastric cancer, papillary thyroid carcinoma, renal cancer [12, 13]. Moreover, in HCC progression, miR-363 has been found to be downregulated and inhibits the tumorigenesis via directly targeting specificity protein [14].

LncRNA NNT-AS1 is a novel identified lncRNA, which is located at 5p12 with 3 exons. In this study, we found that NNT-AS1 expression was upregulated in HCC tissues and cells, and NNT-AS1 knockdown suppressed HCC cells proliferation and invasion. Moreover, NNT-AS1 was negatively correlated with miR-363, acting as ceRNA in the modulation.

## RESULTS

### NNT-AS1 was up-regulated in HCC tissues and correlated with poor prognosis

**Figure 1:**
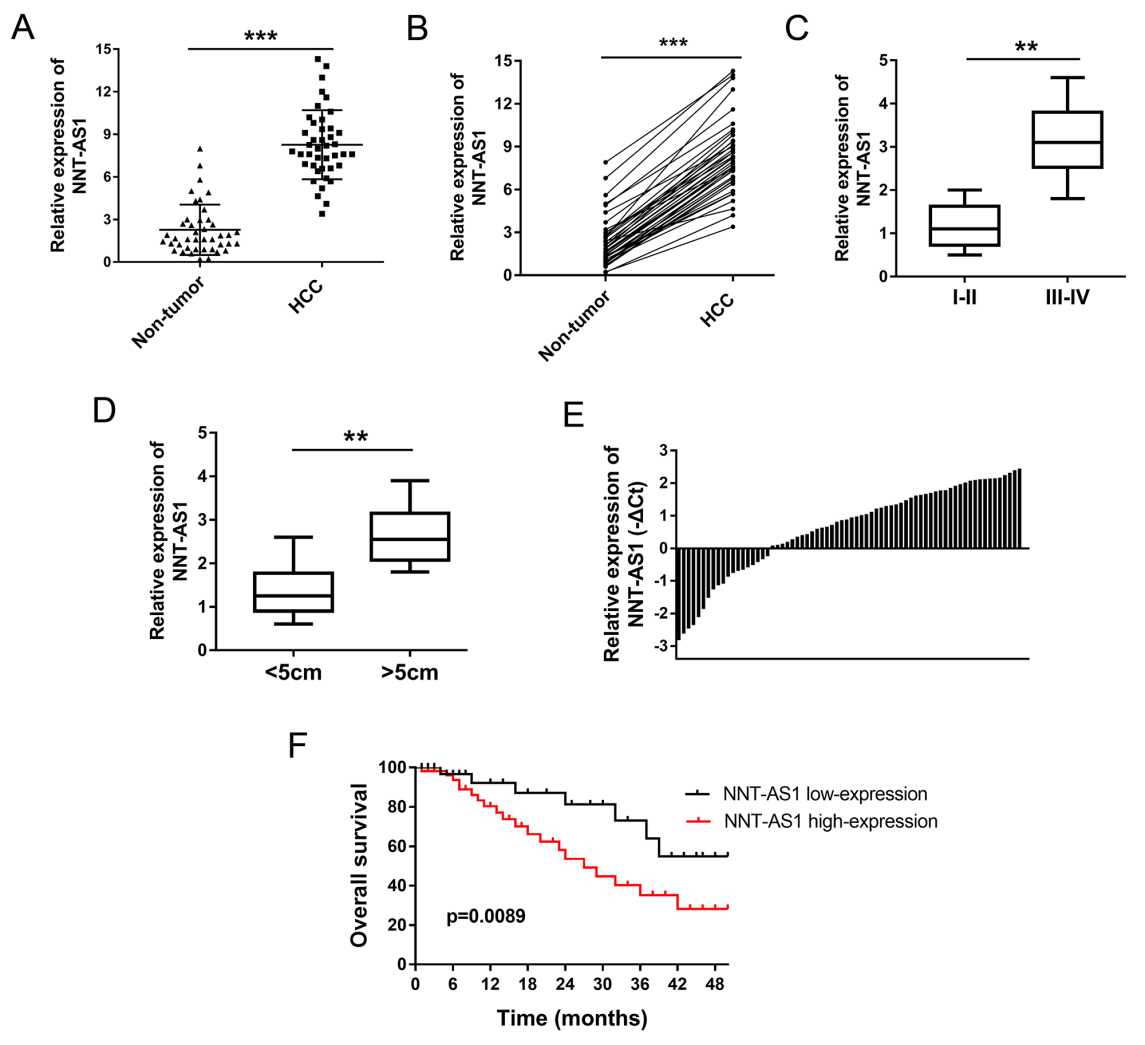
NNT-AS1 was up-regulated in HCC tissues and correlated with poor prognosis **(A)** NNT-AS1 expression in HCC tissues and adjacent normal tissues were detected using RT-PCR. **(B)** The corresponding expression in HCC tissues and matched adjacent normal tissues. **(C)** NNT-AS1 expression in HCC tissues with advanced clinical stage (III-IV) and initial clinical stage (I-II phase). **(D)** Expression of NNT-AS1 in larger tumor volume (>5 cm) and smaller tumor volume (<5 cm). **(E)** Expression levels of NNT-AS1 in 42 pairs of HCC tissues were measured by real-time PCR and calculated using log_2_ method normalized relative to GAPDH. According the NNT-AS1 median expression, all samples were divided into two groups, including a higher NNT-AS1 expression group (n=23) and a lower NNT-AS1 expression group (n=19). **(F)** Overall survivals calculated by Kaplan-Meier curves and log-rank test (p=0.0089). Data are presented as the mean ± SD. ***P<0.001, **P<0.01 compared to control group.

Expression of NNT-AS1 was detected in 42 cases of HCC tissues and paired adjacent normal tissues using RT-PCR. The relationship between NNT-AS1 expression and clinicopathological characteristics of HCC patients was shown in Table 1. Results showed that NNT-AS1 expression was found to be significantly higher in HCC tissues than that of adjacent normal tissues (Figure 1A). Besides, the expression was almost upregulated in all HCC tissues and matched adjacent normal tissues (Figure 1B). Moreover, expression of NNT-AS1 was significantly higher in advanced clinical stage (III-IV phase) patients than that in initial clinical stage (I-II phase) (Figure 1C). Expression of NNT-AS1 was higher in larger tumor volume (>5 cm) than that in smaller tumor volume (<5 cm) (Figure 1D). According the NNT-AS1 median expression, 42 cases of HCC tissues samples were divided into two groups, including a higher NNT-AS1 expression group (n=23) and a lower NNT-AS1 expression group (n=19) (Figure 1E). Overall survivals calculated by Kaplan-Meier curves and log-rank test showed that HCC patients with high NNT-AS1 levels had poor prognosis than that with low NNT-AS1 level (p=0.0089) (Figure 1F). Overall, results indicated that the enhanced expression of NNT-AS1 was closely correlated with poor prognosis of HCC patients.

**Table 1:** Relationship between NNT-AS1 expression and clinicopathological characteristics of HCC patients

Variable		N (N=42)	NNT-AS1 expression		P
			Low (N=19)	High (N=23)	
Gender	Male	28	13	15	0.535
	Female	14	6	8	
Age (years)	<50	23	10	13	0.631
	≥50	19	9	10	
Tumor size	<5cm	15	14	1	0.076*
	≥5cm	27	5	22	
Serum AFP (ng/mL)	<400	13	13	0	0.231
	≥400	29	6	23	
Histological grade	Well/moderately	18	15	3	0.564
	Poorly/others	24	4	20	
Venous infiltration	Absent	17	16	1	0.068
	Present	25	3	22	
Edmondson-Steiner grading	I-II	23	18	5	0.045*
	III-IV	19	1	18	
Lymph node metastasis	Yes	27	5	22	0.143
	No	15	14	1	
TNM stage	I-II	10	10	0	0.021*
	III-IV	32	9	23	

*P<0.05 represents statistical differences. AFP, alpha-fetoprotein; TNM, tumor-node-metastasis.

### NNT-AS1 promoted the proliferation of HCC cells *in vitro*

**Figure 2:**
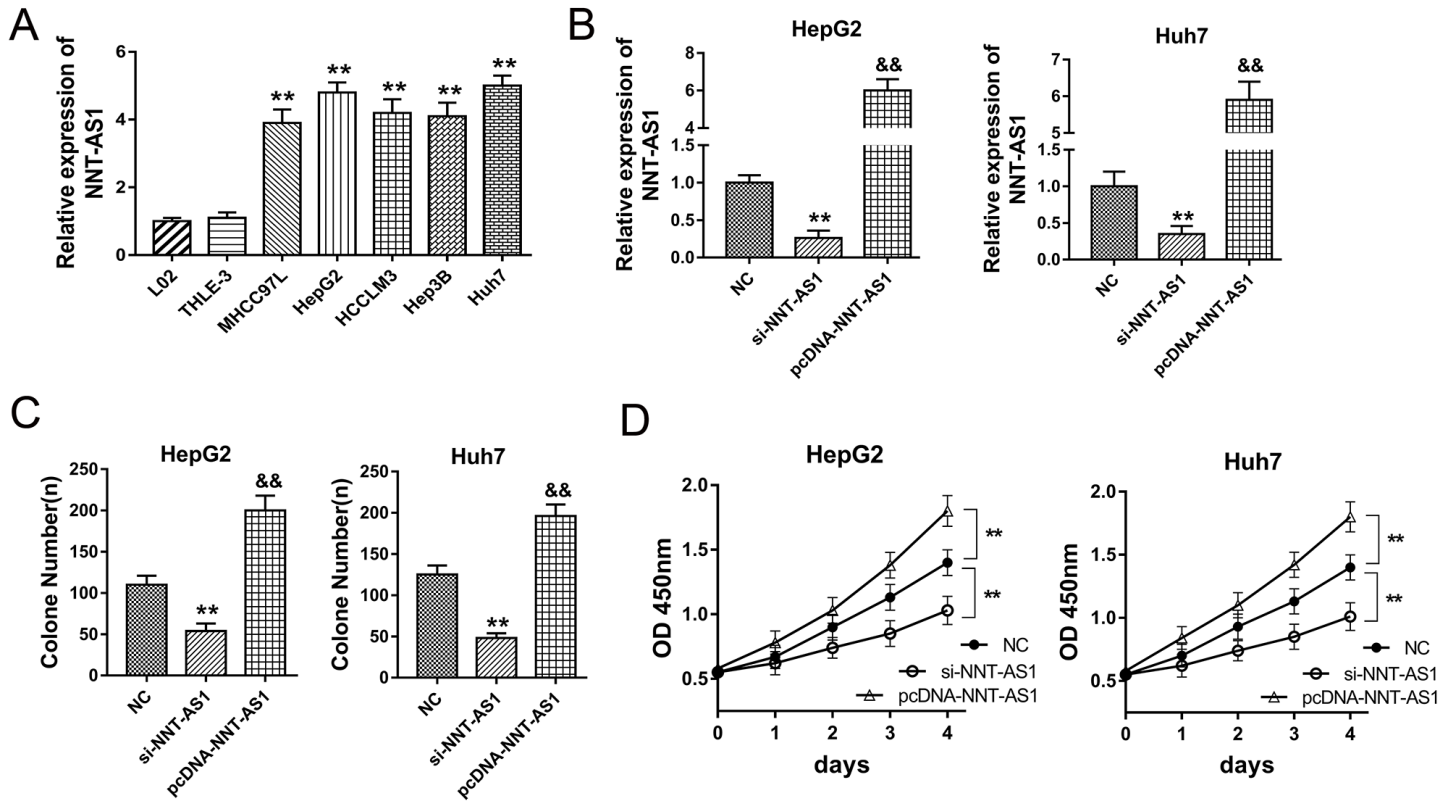
NNT-AS1 promoted the proliferation of HCC cells *in vitro* **(A)** NNT-AS1 expression in human HCC cell lines (MHCC97L, HepG2, HCCLM3, Hep3B and Huh7) and normal liver cell lines (THLE-3, L-02) measured by RT-PCR. **(B)** NNT-AS1 expression in HepG2 and Huh7 cells transfected with si-NNT-AS1 or plasmid vector. **(C)** Colony formation assay showed the proliferation vitality of HCC cells. **(D)** CCK-8 assay showed the absorbance of HepG2 and Huh7 cells at 450 nm. Data are presented as the mean ± SD. **P<0.01, *P<0.05 compared to NC group. &&P<0.01, &P<0.05 compared to si-NNT-AS1 and NC group.

NNT-AS1 had been tested to be up-regulated in HCC tissue and associated with poor prognosis of HCC patients. *in vitro*, NNT-AS1 expression was measured using RT-PCR and found to be significantly up-regulated in HCC cells (Figure 2A). To investigate the role of NNT-AS1 on HCC cells’ tumor characteristic, gain- and loss-of-function experiments were performed in HepG2 and Huh7 cells. In HepG2 and Huh7 cells, NNT-AS1 expression was up-regulated or down-regulated when transfected with si- NNT-AS1 or plasmid vector (Figure 2B). Colony formation assay and CCK-8 assay showed that enhanced NNT-AS1 expression promoted the proliferation vitality of HCC cells, while NNT-AS1 knockdown suppressed the proliferation (Figure 2C, 2D). In summary, our data indicated that NNT-AS1 promoted the proliferation of HCC cells *in vitro*, acting as an oncogenic lncRNA in the HCC tumorigenesis.

### NNT-AS1 alleviated the cell cycle and apoptosis of HCC cells *in vitro*

**Figure 3:**
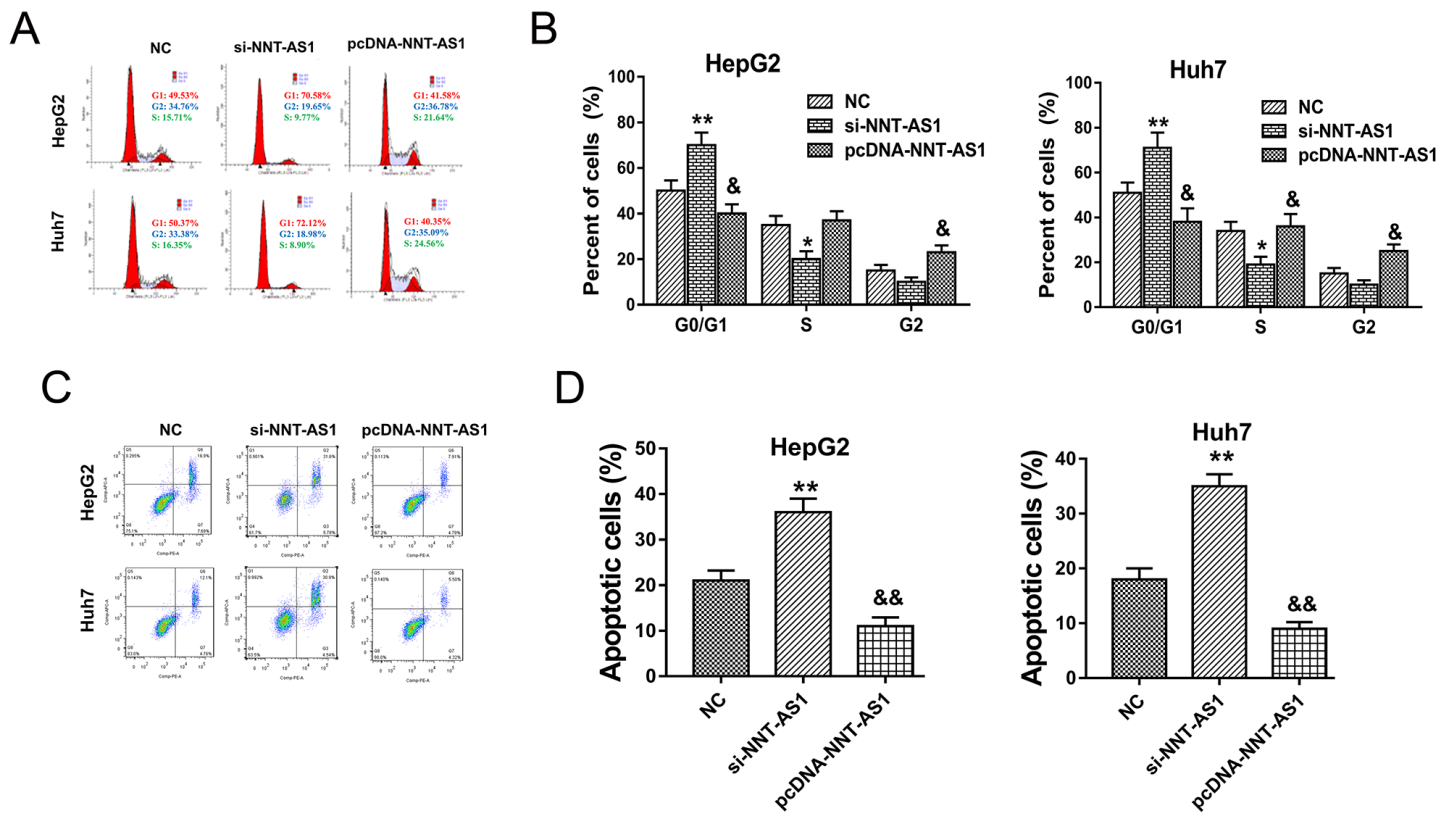
NNT-AS1 alleviated the cell cycle and apoptosis of HCC cells *in vitro* **(A)** Cell cycle arrest detected by flow cytometry showed the cellular distribution in diverse phase. **(B)** The quantitative value of cell distribution in G0/G1, S and G2/M phase. **(C)** Apoptosis detected by flow cytometry showed the apoptotic cell rate in HepG2 and Huh7 transfected with si-NNT-AS1 or plasmid vector. **(D)** The quantitative apoptotic cell rate (%). Data are presented as the mean ± SD. **P<0.01, *P<0.05 compared to NC group. &&P<0.01, &P<0.05 compared to si-NNT-AS1 and NC group.

In prior experiments, NNT-AS1 acted as an oncogenic lncRNA to promote HCC cells proliferation *in vitro*. In further research, the role of NNT-AS1 on HCC cells cell cycle and apoptosis was detected using flow cytometry. Results showed that NNT-AS1 knockdown induced the cell cycle arrest at G0/G1 phase and promoted the apoptosis, while NNT-AS1 enhanced expression alleviated the cycle arrest at G0/G1 phase and decreased the apoptotic cell rate compared to control group (Figure 3A-3D). Overall, results of flow cytometry showed that NNT-AS1 could alleviate the cell cycle arrest and apoptosis of HCC cells *in vitro*.

### NNT-AS1 knockdown inhibited the HCC tumor growth *in vivo*

**Figure 4:**
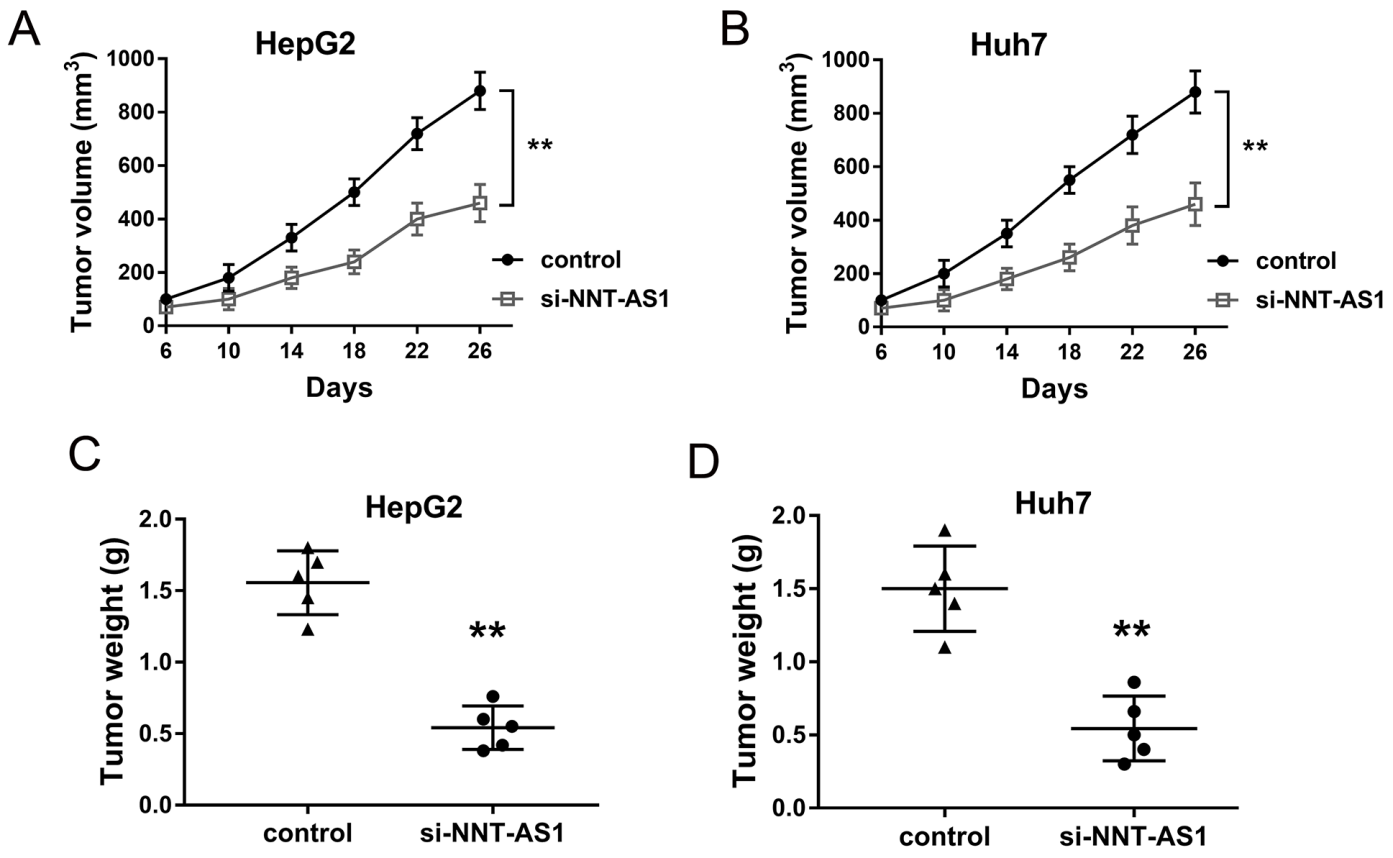
NNT-AS1 knockdown inhibited the HCC tumor growth *in vivo* **(A)** Tumor volume was measured every four days after injected with HepG2 cells transfected sh-NNT-AS1 plasmid. **(B)** Tumor volume injected with Huh7 cells transfected si-NNT-AS1 plasmid. **(C, D)** The neoplastic tumor weight was measured after mice sacrifice. Data are presented as the mean ± SD. **P<0.01 compared to control group.

In order to investigate the role of NNT-AS1 on HCC tumor formation and growth *in vivo*, HepG2 and Huh7 cells transfected with si-NNT-AS1 and injected into subcutaneous of nude mice. Results showed that NNT-AS1 knockdown transfected with sh-NNT-AS1 could significantly decrease the tumor volume compared with control group (Figure 4A, 4B). After sacrificed, the neoplastic tumor was resected and the weight in NNT-AS1 knockdown group was significantly smaller than that in control group (Figure 4C, 4D). Therefore, results indicated that NNT-AS1 knockdown could inhibit the HCC tumor growth *in vivo*.

### NNT-AS1 directly bound miR-363 at 3’-UTR

**Figure 5:**
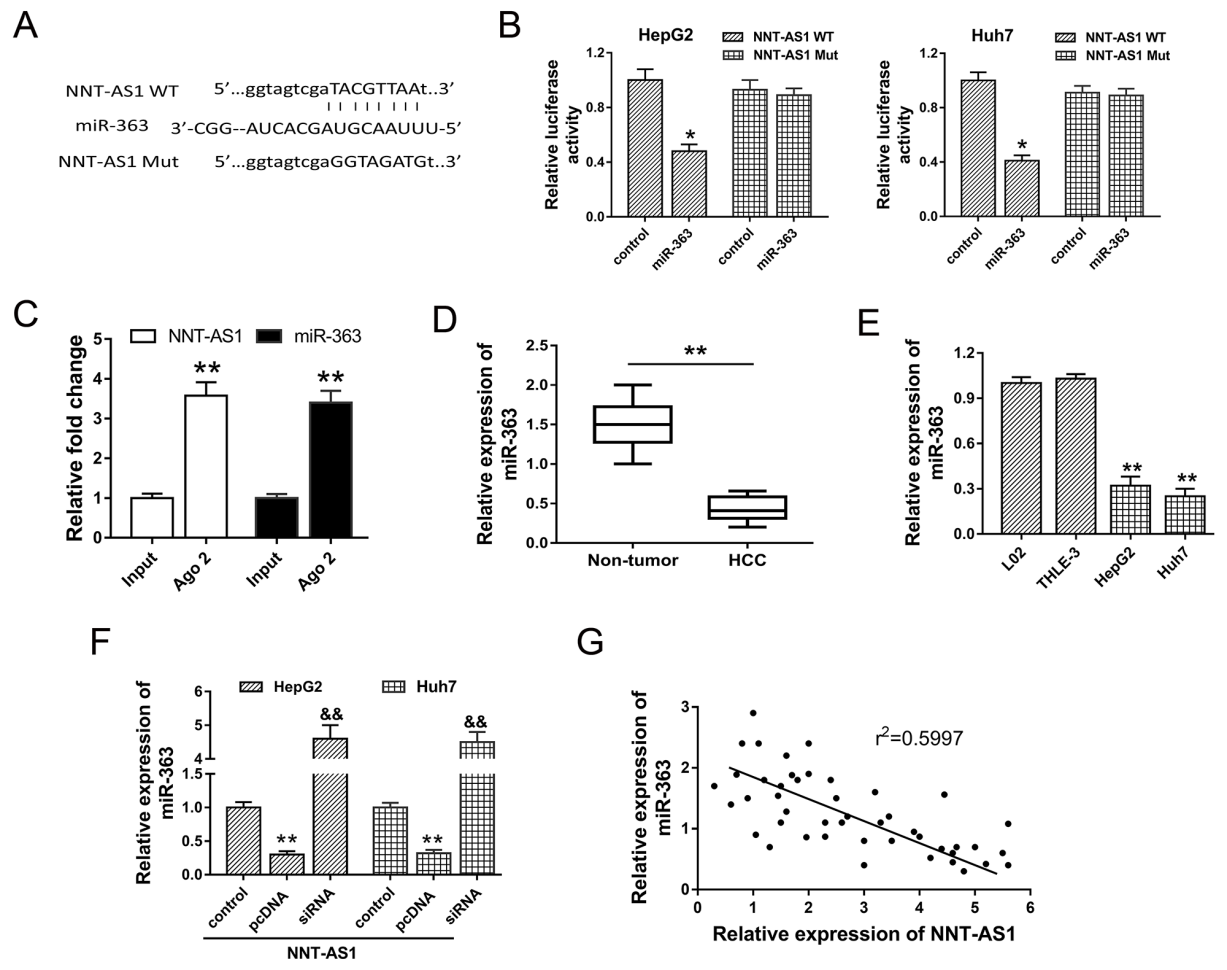
NNT-AS1 directly bound miR-363 at 3’-UTR **(A)** The putative complementary binding sequence of NNT-AS1 3’-UTR and miR-363 according to starBase, TargetScan and miRBase. **(B)** Luciferase reporter assay showed the luciferase vitality in HepG2 and Huh7 cells transfected with NNT-AS1 (wild type or mutant) and miR-363 (mimics or control blank). **(C)** RIP assay showed the enrichment of NNT-AS1 and miR-363 Ago2-containing beads. **(D)** Expression level of miR-363 assessed in HCC tissue samples and adjacent noncancerous tissue. **(E)** MiR-363 expression in HCC cells. **(F)** MiR-363 expression in HepG2 and Huh7 cells transfected with NNT-AS1 plasmid vector or siRNAs. **(G)** Pearson’s correlation analysis showed the correlations between NNT-AS1 and miR-363 expression in 42 cases of HCC tissue (r^2^=0.5997, P=0.0185). Data are presented as the mean ± SD. &&P<0.01, **P<0.01, *P<0.05 compared to control group.

Bioinformatics prediction program indicated that NNT-AS1 contained complementary binding sequence with miR-363 at 3’-UTR (Figure 5A). Luciferase reporter assay validated the decreasing of luciferase vitality in HepG2 and Huh7 cells transfected with NNT-AS1 wild type and miR-363 mimics, suggesting the binding within NNT-AS1 3’-UTR and miR-363 (Figure 5B). RIP assay showed that NNT-AS1 and miR-363 were significantly enriched in Ago2-containing beads compared to input group (Figure 5C). Expression level of miR-363 assessed in HCC tissue samples showed that miR-363 was down-regulated in HCC tissue compared with adjacent noncancerous tissue (Figure 5D). Similarly, miR-363 expression in HCC cells was significantly decreased (Figure 5E). Furthermore, in HepG2 and Huh7 cells transfected with NNT-AS1 plasmid vector or siRNAs, miR-363 expression was significantly down-regulated or up-regulated, showing the opposite relationship within NNT-AS1 and miR-363 (Figure 5F). Pearson’s correlation analysis and long rank analysis showed the negatively association within the expression of NNT-AS1 and miR-363 in 42 cases of HCC tissues (Figure 5G). In summary, results revealed the direct binding within NNT-AS1 and miR-363, suggesting the potential miRNA ‘sponge’ role of NNT-AS1 for miR-363.

### NNT-AS1 promoted CDK6 expression through miR-363

**Figure 6:**
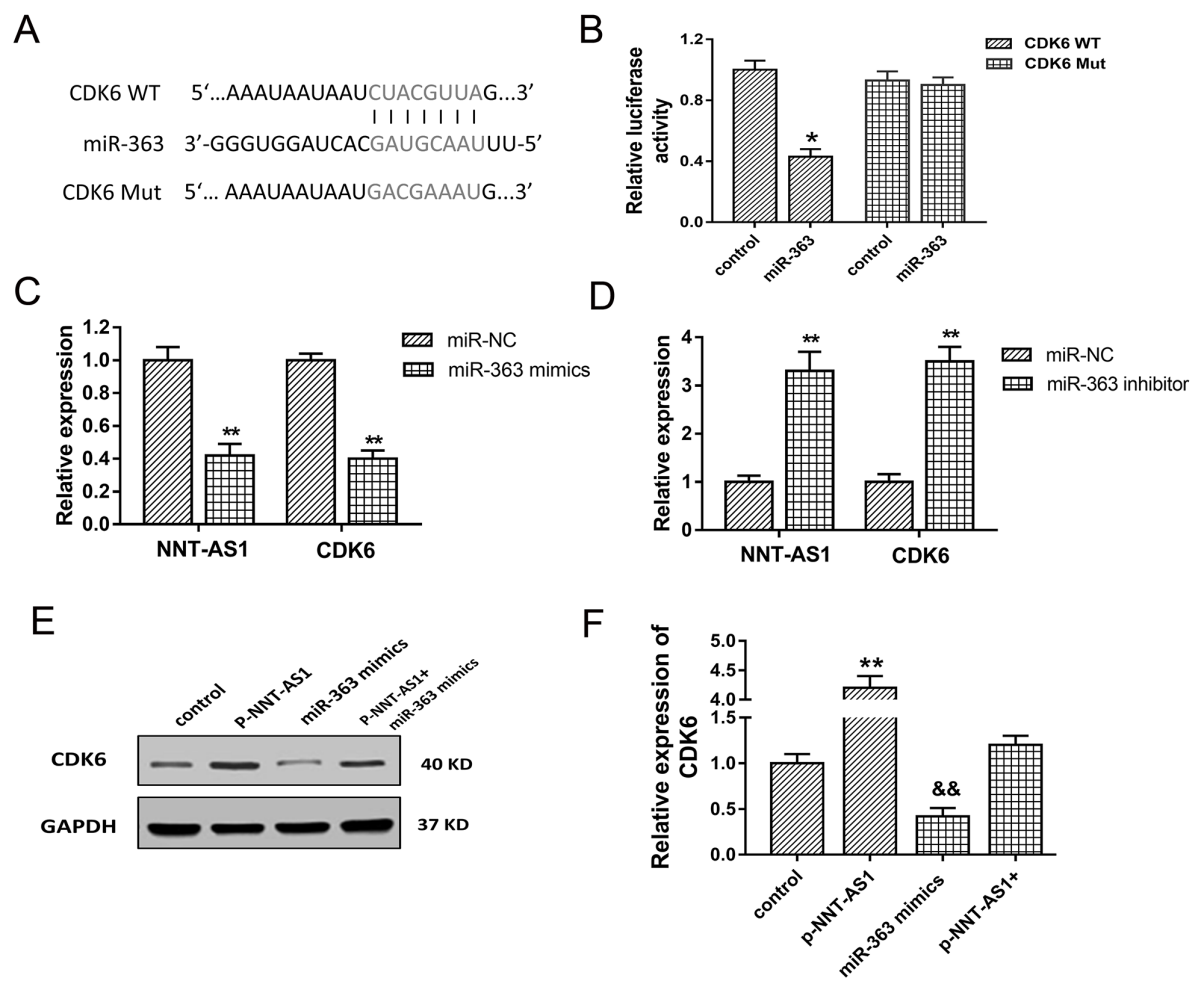
CDK6 was target gene of miR-363 **(A)** The putative complementary binding sequence of CDK6 3’-UTR and miR-363 according to TargetScan and miRBase. **(B)** Luciferase reporter assay showed the luciferase vitality in HepG2 cells transfected with CDK6 (wild type or mutant) and miR-363 (mimics or control blank). **(C)** NNT-AS1 and CDK6 mRNA expression in HepG2 cells transfected with miR-363 mimics. **(D)** NNT-AS1 and CDK6 mRNA expression in HepG2 cells transfected with miR-363 inhibitor. **(E)** Western blots of CDK6 in HepG2 cells transfected with pcDNA-NNT-AS1 or/and miR-363 mimics. **(F)** Quantitative value of CDK6 calculated by fold change. Data are presented as the mean ± SD. &&P<0.01, **P<0.01, *P<0.05 compared to control group.

To investigate the role of NNT-AS1/miR-363 in the cell cycle regulation, bioinformatics analysis was performed to discover the target gene of miR-363 in HCC tumorigenesis. Results indicated that CDK6 was one of target mRNA for miR-363 with 7 binding sites (Figure 6A). Luciferase reporter assay showed that miR-363 directly targeted CDK6 3’-UTR (Figure 6B). In HepG2 cells transfected with miR-363 mimics, NNT-AS1 and CDK6 mRNA expression were both decreased compared with negative controls group (Figure 6C). On the contrary, in HepG2 cells transfected with miR-363 inhibitor, NNT-AS1 and CDK6 mRNA expression were both increased compared with controls group (Figure 6D). Western blots showed that enhanced NNT-AS1 expression increased the CDK6 expression, and NNT-AS1 lower-expression decreased the CDK6 expression. Moreover, co-transfection of pcDNA-NNT-AS1 and miR-363 mimics recovered the CDK6 expression compared with control group (Figure 6E, 6F). In summary, results indicated that CDK6 was target gene of miR-363 and NNT-AS1 promoted CDK6 expression through miR-363.

## DISCUSSION

Increasing evidences have indicated that lncRNAs participate in a wide range of cellular processes, including regulation of epigenetic signatures, gene expression and proliferation [15, 16]. Especially in HCC, a growing number of lncRNAs have been reported to participate in the occurrence, progression and metastasis, for example MALAT1, MEG3 and UCA1 [17-20]. In present study, we provide evidence that NNT-AS1 exerts oncogenic role in the HCC progression and metastasis via miR-363/CDK6 axis.

LncRNAs are a type non-coding RNA (ncRNA) without protein translation capacity. With regard to tumorigenesis, lncRNAs participate in transcriptional or post-transcriptional level regulation according to the cellular localization of nucleus or cytoplasm [21]. Emerging evidences have manifested that lncRNAs play vital role in all the carcinogenesis process, including differentiation, proliferation, invasion and metastasis [22, 23]. Taking HCC as an example, lncRNA lnc-EGFR links an immunosuppressive state to cancer by promoting Treg cell differentiation, and promotes hepatocellular carcinoma immune evasion [24]. LncRNA TP73-AS1 modulates HCC cell proliferation via miR-200a/ HMGB1/RAGE pathway, being inversely correlated with miR-200a and positively correlated with HMGB1 and RAGE [25].

LncRNA Nicotinamide Nucleotide Transhydrogenase-antisense RNA1 (NNT-AS1) is a novel identified lncRNA. In our study, our team found that NNT-AS1 expression was significantly up-regulated in 35 pairs of HCC tissue and adjacent normal tissue, as well as in HCC cells. Moreover, the overexpression of NNT-AS1 is closely correlated with advanced clinical stage (III-IV phase), larger tumor volume (>5 cm) and poor prognosis (p=0.0089). *In vitro* and *vivo* assay, NNT-AS1 has been confirmed to accelerate the proliferation and tumor growth, suppress the apoptosis for HCC. In summary, our results conclude that NNT-AS1 acts as an oncogenic lncRNA in the HCC tumorigenesis.

A lot of researches have illustrated the potential role of lncRNA in carcinogenesis [26, 27]. In colorectal cancer, NNT-AS1 has been tested to participate in the progression of colorectal cancer cells via regulating proliferation, migration, and invasion by MAPK/Erk signaling pathway and epithelial-mesenchymal transition (EMT) [28]. Moreover, NNT-AS1 expression in cervical cancer tissues is overexpressed compared with adjacent non-tumor tissues, and the overexpression of NNT-AS1 is positively associated with advanced FIGO stage, lymph node metastasis, depth of cervical invasion and poorer overall survival [29]. CCAT2 is associated with poor prognosis in hepatocellular carcinoma and enhanced the EMT through regulating vimentin, E-cadherin and transcription factor snail2 expression [18]. In present study, we revealed the oncogenic role of NNT-AS1 on HCC cells proliferation and metastasis, which is the first time to report its biologic role on HCC.

In pre-experiments, we found that NNT-AS1 was mainly located in cytoplasm. Therefore, we refer the canonical miRNA ‘sponge’ mechanism to investigate the underlying regulation of NNT-AS1 on HCC. With the help of bioinformatics analysis and online tools, we predicted that miR-363 contained complementary binding site targeting NNT-AS1 3’-UTR, which was confirmed by luciferase reporter assay. Admittedly, the major approach that lncRNA exerted physiological function is to act as miRNA ‘sponge’ to decrease target miRNAs’ abundance to indirectly modulate functional gene or mRNAs function. Furthermore, we predicted and validated that CDK6 was one of the target gene of miR-363. Comprehensive RT-PCR and western blot assay revealed that miR-363 expression was negatively correlated with CDK6 mRNA and NNT-AS1 expression. As is well-known, CDK protein family (including CDK6) is the core molecular in the tumor cell cycle regulation, accompanying with cyclin and cyclin-dependent-kinase inhibitor (CKI). Thus, combined with the verified oncogenic role of NNT-AS1 in HCC, we could conclude that NNT-AS1 exert the tumor-promoting role via miR-363/CDK6 axis.

The competing endogenous RNA (ceRNA) mechanism is a vital way for lncRNAs to regulate the huge number of physiological and pathological process. Besides, miRNA ‘sponge’ is another description for ceRNA, absorbing the targeting miRNAs linking sponge. For example, lncRNA TUG1 promotes gastric cancer cells proliferation and invasion in via negatively modulating miRNA-145-5p [30]. In HCC, lncRNA XIST regulates PTEN to promote proliferation and invasion expression by sponging miR-181a [31]. Our study revealed that NNT-AS1 promoted HCC progression and tumor growth via sponging miR-363, which might illustrate the regulatory way for HCC. CDK6 acts as the target mRNA of miR-363, which is a vital molecular on cell cycle progression regulation. Furthermore, *in vitro* cellular assay, loss-of-function experiments indicated that NNT-AS1 knockdown induced the cell cycle arrest at G0/G1 phase and promoted the apoptosis. Therefore, our results of summarize evidence support the conclusion that NNT-AS1 modulates the HCC cell progression through positively regulating CDK6 to promote the HCC tumorigenesis. Thus, via cycle promoting, NNT-AS1 promotes the proliferation of HCC cells *in vitro*, acting as an oncogenic lncRNA in the HCC tumorigenesis. NNT-AS1/miR-363/ CDK6 axis provides a novel insight for the pathological process.

In summary, our study and results summarize that NNT-AS1 functions as an oncogenic lncRNA for HCC tumorigenesis through miR-363/CDK6 axis, which might be a useful diagnostic index and prognostic biomarker, providing a novel therapeutic target for human HCC.

## MATERIALS AND METHODS

### Tissue collection

All tissue samples were collected with written informed consent in accordance with the requirements of the Research Ethics Committee of Xiangya Hospital of Central South University. Total 42 cases of HCC tissues and paired adjacent normal tissues were obtained from patients who had undergone surgery without chemotherapy or radiotherapy at the Xiangya Hospital of Central South University between Jan 2016 and Dec 2016. Tumor tissue and normal adjacent specimens were immediately snap-frozen in liquid nitrogen and stored at -80°C as soon as excised. All tissues were respectively confirmed by two experienced histopathologist.

### Cells culture and interfering RNA transfection

Human HCC cell lines (MHCC97L, HepG2, HCCLM3, Hep3B and Huh7) and normal liver cell lines (THLE-3, L-02) were purchased from Cell Bank of the Shanghai Branch of Chinese Academy of Sciences (Shanghai, China). All cells were cultured in Dulbecco’s Modified Eagle’s Medium (DMEM, Gibco, USA) containing 10% fetal bovine serum (FBS, Hyclone, USA) and 1% penicillin/streptomycin. The culture condition was kept at 5% CO_2_ at 37 °C.

NNT-AS1 specific small interfering RNA (si-NNT-AS1) targeting NNT-AS1 and negative control were synthesized by RiboBio (Guangzhou, China). Plasmid vectors (NNT-AS1 and empty vector) for transfection were extracted using a DNA Midiprep kit (Qiagen, Hilden, Germany). The full-length complementary DNA of NNT-AS1 was synthesized and cloned into the pcDNA3.1 vector (Invitrogen, Carlsbad, Calif, USA) according to the manufacturer’s instructions. Cell transfections were conducted using the Lipofectamine 2000 transfection reagent according to manufacturers’ instructions.

### Real-time polymerase chain reaction (PCR)

Total RNA was extracted from tumor tissue and adjacent non-tumorous or cells using Trizol reagent (Invitrogen, Carlsbad, Calif, USA). Total RNA (1 μg) was reverse-transcribed to cDNA using High Capacity cDNA Reverse Transcription Kit (Applied Biosystems, Darmstadt, Germany). RT-PCR was performed using SYBR Green real-time PCR kit (TaKaRa, Dalian, China). Glyceraldehyde 3-phosphate dehydrogenase (GAPDH) acted as the internal reference for normalization expression. All the primers were shown as follows: NNT-AS1, forward, 5’-ACGTGCAGACAACATCTACCT-3’, reverse, 5’-TACAACACCTTCCCGC AT-3’; GAPDH, forward, 5’-CCCATCACCATCTTCCAGGAG-3’, reverse 5’-GTTGTCATGGATGACCTTGGC-3’. Relative expression level was calculated with 2^−ΔΔCt^ method.

### Cell proliferation assays

The Cell Counting Kit-8 (CCK-8, Dojindo, Japan) proliferation assay was performed with HepG2 and Huh7 cells according to the manufacturer’s protocol. Logarithmically growing HCC cell lines (5×10^3^ cells/well) were inoculated into 96-well culture plates. CCK-8 assay solution (10μL) was added to each well after incubation for several days. Afterwards, enzyme immunoassay analyzer (Thermo Fisher Scientific, Inc., USA) was used to measure the optical density (OD) at 450nm.

### Colony formation assay

HCC cells (HepG2 and Huh7) were plated in 6-well culture plates at a density of 100 cells per well. After incubation for 14 days at 37°C, cells were fixed with methanol and stained with 0.2% crystal violet and then washed three times with phosphate buffered saline (PBS). Finally, the visible colonies consisting of >50 cells were manually counted.

### Cycle analysis and apoptosis assays

Flow cytometric analysis was performed to detect the cycle and apoptosis analysis. Briefly, HCC cells were transiently transfected with siRNA or pcDNA3.1-NNT-AS1, and harvested after 48h of transfection by trypsinization. For apoptosis analysis, cells were stained with Annexin V-FITC Apoptosis Detection Kit (BD Bio sciences, Franklin Lakes, NJ, USA) and incubated at room temperature for 15 mins in the dark. Then, FITC and PI fluorescence were analyzed by FACSCalibur Flow Cytometer (BD Bioscience, USA) and BD CellQuest software (BD Bioscience, USA). For cell cycle analysis, HCC cells were stained with Cell Cycle Detection Kit (Becton Dickinson, Heidelberg, Germany) according to the manufacturer’s instructions. The cell cycle analysis was detected by flow cytometry.

### Luciferase reporter assay

Luciferase assays were performed using the luciferase reporter assay system (Promega, Madison, WI, USA) according to the manufacturer instructions. Briefly, wild-type (WT) or mutated-type (Mut) of NNT-AS1 or CDK6 3’-UTR sequence containing the miR-363 targeting sites were inserted into pGL3 promoter vector (Invitrogen, Carlsbad, Calif, USA) to construct pGL3-luc-NNT-AS1. HCC cells (HepG2 and Huh7) were seeded into 96-well plates and transfected with of pGL3-luc-NNT-AS1 (50 ng) and Renilla luciferase (5 ng) and miR-363 mimics or NC (5 pmol) using the Lipofectamine 2000 according to the manufacturer’s instructions. After transfection of 48 h, the luciferase activity was detected by dual luciferase assay (Promega, Madison, WI, USA), normalized with Renilla luciferase activity. All experiments were performed in triplicate.

### RNA immunoprecipitation (RIP) assay

RIP assay was performed as previously described to validate the interaction within NNT-AS1 and miR-363 using EZ-Magna RIP RNA-binding protein immunoprecipitation kit (Millipore, Billerica, MA, USA) following the manufacturer’s instructions. Briefly, HepG2 cells at 80-90% confluency were lysed in RIP lysis buffer, and incubated with the RIP buffer containing magnetic beads coated with anti-human argonaute 2 (Ago2) antibodies (Millipore). Isotype-matched immunoglobulin G (IgG) was used as a negative control. Then, RNAs were extracted and subjected to RT-qPCR.

### Western blot analysis

Total protein was extracted from HCC cells using RIPA lysis buffer (Invitrogen, Carlsbad, Calif, USA). Briefly, equal amounts of A quantity of approximately 30 μg of whole cell lysates per lane were separated using 10% SDS-PAGE and transferred on to immobilon PVDF membranes (Millipore, Corporation, Billerica, MA, USA). PVDF membranes were incubated with the primary antibodies of CDK6 (Abcam, 1:2000 dilution) and anti-GAPDH (Santa Cruz, 1:1,000 dilution). Blots were incubated with goat anti-rabbit secondary antibody conjugated to HRP (Abcam, 1:1000 dilution). PVDF membranes were visualized by a standard enhanced chemiluminescence procedure (Millipore, Billerica, MA, USA). The signals were analyzed using Image Lab software (Bio-Rad Laboratories, Inc., Hercules, CA). The band densities of each sample were normalized to the GAPDH band.

### Tumor xenograft model in nude mice

This research was approved by the Institutional Animal Care Committee of Xiangya Hospital of Central South University. Male BALB/C nude mice (20 mice, 4-6 weeks) were purchased from the Slac Laboratory Animal Center (Shanghai, China) and maintained under specific pathogen-free conditions. All animals were housed in micro-isolator cages with free access to food and water according to the Guide for the Care and Use of Laboratory Animals. Then, 150 μl suspension of HepG2 and Huh7 cells (3×10^6^ per 100 ml) transfected with sh-NNT-AS1 or sh-NC were respectively subcutaneously injected into the back of nude mice. Tumour size was measured every 4 days starting 6^th^ days of injection. Tumour volume was harvested after sacrifice and calculated using a simplified equation (0.5 × length × width^2^).

### Statistical analysis

All experiments were performed at least three times. All data are represented as the mean ± standard deviation (SD). Pearson’s correlation coefficient was used to measure linear correlations between two variables. The two groups’ differences were analyzed using Student’s t-test or one way ANOVA. 0.05 were considered to indicate statistical significance.
